# A Rapidly-progressing Outbreak of Cholera in a Shelter-home for Mentally-retarded Females, Amta-II Block, Howrah, West Bengal, India

**DOI:** 10.3329/jhpn.v30i1.11290

**Published:** 2012-03

**Authors:** Subhransu Sekhar Datta, R. Ramakrishnan, Manoj V. Murhekar

**Affiliations:** Field Epidemiology Training Programme, National Institute of Epidemiology (Indian Council of Medical Research), Chennai, India

**Keywords:** Cholera, Cohort studies, Disease outbreaks, Retrospective studies, Risk factors, *Vibrio cholerae*, India

## Abstract

On 13 May 2010, a cluster of diarrhoeal disease cases was reported among the inmates of a shelter-home for mentally-retarded females in Parbaksi village of Howrah district in West Bengal, India. The outbreak was investigated to identify the aetiological agent and source of infection and to propose recommendations. A suspected case of cholera was defined as an acute onset of >3 loose watery stools in a female resident of the shelter-home since 1 May 2010. The demographic and clinical details were collected from the suspected case-patients, and the outbreak was described by time, place, and person. A retrospective cohort study was conducted to identify the risk factors associated with the illness. Of the 101 inmates, 91 (90%) developed diarrhoea, and three patients died (case fatality−3%). Four of the five stool specimens were positive for *Vibrio cholerae* O1 Ogawa. Drinking of water from the pond-connected tubewell (adjusted odds ratio=25.7, 95% confidence interval 2.7-236.4) was associated with the illness. Relocation of the pond-connected tubewell away from the groundwater tubewell, colour-coding of the tubewells meant for drinking purposes, and regular disinfection of the tubewells were recommended.

## INTRODUCTION

Cholera is an acute infection caused by *Vibrio cholerae* serogroup O1 and O139 (1). Human beings are the main reservoir of *V. cholerae*, and the disease is transmitted through contaminated food or water and from person to person by direct faeco-oral contamination. The disease is characterized by the sudden onset of profuse painless watery diarrhoea, often accompanied with vomiting, which can rapidly lead to severe dehydration and cardiovascular collapse (1). Cholera continues to be an important health problem in India. During 1997-2006, 68 outbreaks of cholera were reported from the country, affecting more than 200,000 persons, with 823 deaths. Nearly one-fourth of the outbreaks and 42% of the deaths occured in West Bengal (2).

On 13 May 2010, the Medical Officer of Amta-II Block in Howrah district informed the district health authorities about a cluster of diarrhoeal disease cases, with three deaths in a shelter-home run by a non-governmental organization (NGO) at Parbaksi village. On further enquiry, we came to know that all the case-patients (including 3 deaths) were females and were admitted to a nearby rural hospital with predominant clinical features of watery stool, lower-abdominal pain, and vomiting with or without dehydration. We initiated investigations on the same day to (a) assess the magnitude of the outbreak, (b) identify the aetiologic agent and source of infection, and (c) initiate control and preventive measures.

## MATERIALS AND METHODS

### Descriptive epidemiology

We defined a suspected case of cholera as an acute onset of watery stools in a resident of the shelter-home of Parbaksi village, Howrah, since 1 May 2010. The demographic and clinical details of the suspected case-patients were collected from the hospital-records and denominator data from the records of the shelter-home. We drew a spot-map, constructed an epidemic curve (Fig.), and calculated the attack rates by age-group. A few responsive case-patients and shelter-home staff were interviewed to identify the possible common source of infection to generate a hypothesis.

### Laboratory examination

We collected stool samples from five case-patients and water samples from all the tubewells and the unguarded pond of the shelter-home. All the specimens were transported in cold-chain conditions to the School of Tropical Medicine (STM) and National Institute of Cholera and Enteric Diseases (NICED), Kolkata, for microbiological examination.

### Environmental investigations

We inspected the shelter-home for different sources of water used for drinking, cooking and domestic purposes and for identifying the possible sources of drinking-water contamination. We also inspected all conditions for food-preparation and handling and interviewed the kitchen staff about the storage of drinking-water and clinically examined the food-handlers.

### Analytical epidemiology

Review of the descriptive epidemiological findings led us to suspect that the illness was associated with (a) drinking the tubewell-water connected to the pond and (b) cognitive function of the inmates. To test these hypotheses, we conducted a retrospective cohort study among all the inmates of the shelter for mentally-retarded females. The residents were interviewed to collect information about the source of drinking-water using photographs of different drinking-water sources in the home. To assess the cognitive function of the inmates, we asked questions regarding their orientation in time and space (name of the day of the week and place of their residence**)** and details of their identification. For the women who could not respond on their own, we collected information with the assistance of the caretaker of the shelter-home.

### Analysis of data

We calculated the relative risk (RR) and 95% confidence intervals (CIs) associated with different exposures. We conducted a multivariate analysis and calculated the adjusted odds ratios (AORs) of selected factors and the respective aetiologic fractions in the population. All statistical analyses were conducted using the Epi Info software (version 3.5.1) (Centers for Disease Control and Prevention, Atlanta, Georgia, USA) and the OpenEpi software (version 2) (www.openepi.com).

## RESULTS

### Descriptive epidemiology

The shelter-home comprised a hostel for mentally-retarded females (n=101), two hostels for mentally-normal but destitute and orphan boys (n=71), and a school for these children. We identified 91 case-patients who met the case definition from among the 101 mentally-retarded female residents of the shelter-home (attack rate−90%). Three case-patients died (case-fatality ratio−3%). The attack rate was the highest among individuals aged less than four years and 45-59 years (100%) (Table 1). The first case-patient developed illness on 9 May 2010 and died on 10 May. The outbreak peaked on 13 May. The number of cases began to decrease following the use of packaged drinking-water from the evening of 13 May, temporary closure of the tubewell connected to the pond and disinfection of other groundwater tubewells on 14 May. The shape of the epidemic curve suggested a point-source outbreak (Fig.). None of the inmates in the boys’ hostel and orphanage or none of the staff members of the shelter-home was affected.

**Table 1.** Age-group-specific attack rate of cholera among the inmates of the NGO-run shelter-home at Amta-II Block, Howrah, West Bengal, India, May 2010

Age-group (years)	No. of cases	No. of residents	Attack rate (%)
0-4	5	5	100
5-14	30	34	88
15-29	48	49	98
30-44	6	11	55
45-59	2	2	100
Overall	91	101	90

NGO=Non-governmental organization

### Laboratory investigations

Four of the five stool specimens were culture-positive for *V. cholerae*, serogroup O1, serotype Ogawa on thiosulphate-citrate-bile salt-sucrose (TCBS) agar. The water sample of the pond-connected tubewell contained coliforms but that of the groundwater tubewell was potable. The unguarded pond-water was grossly contaminated.

### Environmental investigations

The shelter-home had two sources of drinking-water: one groundwater tubewell adjacent to the school building for boys and staff members and the other one exclusively for the females residing in the hostel. Besides, there was one tubewell connected to the pond (used for domestic purposes by females) situated next to the groundwater tubewell in the female hostel. We observed that unguarded pond-water was directly used by boys and other staff members for bathing/washing. The kitchen was unhygienic but we did not find any health problems among the food-handlers. We also found that same prepared foods were carried to the dining room of the female hostel for serving the mentally-retarded female inmates, and the remaining members of staff and boys were served in the kitchen itself. In-depth open interviews with a home-caretaker and a few respondent case-patients indicated a possibility of exposure to contaminated pond-connected tubewell water. We also found that the unaffected females had a better cognitive function.

**Fig.**
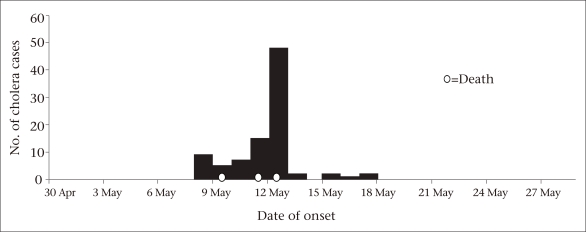
Epidemic curve of cholera outbreak in a shelter-home, Howrah, West Bengal, India, May 2010

### Analytical epidemiology

We included 87 (86%) of the 101 inmates (five children, five hospitalized case-patients, and four deaf and dumb females were excluded) in the cohort study. Of them, 21 (24.1%) responded to the set of questions asked to assess the cognitive function while 66 (75.9%) did not reply. Information about drinking-water sources could be elicited from the 61 (70%) females. Compared to those who reported the groundwater tubewell as the source of drinking-water, women who reported the use of pond-connected tubewell-water were more likely to develop the illness (AOR=25.7, 95% CI 2.7-236.4). Women with poor cognitive function (defined as those who could not give answers to the set of questions) had a higher risk of developing the illness (AOR=5.5, 95% CI 1-30.9). However, the results were not significant (Table 2). The population attributable fraction associated with drinking of pond-connected tubewell-water was 94%.

**Table 2.** Attack of cholera in a shelter-home for mentally-retarded residents according to selected factors, Howrah, India, 2010

Risk factor	Attack of cholera						Odds ratio (95% CI)	Adjusted odds ratio (95% CI)
	Among exposed			Among unexposed				
	No.	Total	%	No.	Total	%		
Drinking of water from pond-tubewell	39	40	97.5	12	21	57.1	29.3 (3.4-254.9)	25.7 (2.7-236.4)
Poor cognitive function	63	66	95.4	14	21	66.6	10.5 (2.4-45.7)	5.5 (1-30.9)

CI=Confidence interval

## DISCUSSION

An explosive outbreak of cholera occurred among the mentally-retarded residents of the NGO-run shelter-home. The outbreak was associated with consumption of water from the pond-connected tubewell. The points supporting our findings included: (a) absence of any cases among the inmates residing in other facilities, who were using the groundwater tubewell for drinking purposes; (b) presence of coliform in pond-connected tubewell water and in the pond; and (c) a significant risk associated with the consumption of water from the pond-connected tubewell, with more than 90% of the cases attributable to this exposure. Vicinity of the pond-connected tubewell meant for domestic use and the groundwater tubewell meant for drinking purpose was the most likely reason for the exposure of the inmates to contaminated drinking-water.

The striking feature of this outbreak was a very high attack rate, affecting more than 90% of the inmates of the shelter-home for the mentally-retarded females. The attack rates observed during most of the outbreaks in the community setting in West Bengal ranged from 0.3% to 7% (3-6). Outbreaks with attack rates exceeding 5% have been reported in refugee settings (7). A very high attack rate observed in this outbreak indicates heavy contamination of the pond-connected tubewell-water.

It was a challenge to collect information about sources of drinking-water from the mentally-challenged individuals. We tried to address this issue using photographs of different drinking-water sources in the shelter-home and seeking assistance from the caretaker for deciding on the cognitive status. We, however, could not collect the exposure information on drinking-water source from 26 (29.9%) inmates. This reduced the sample-size available for the analytical study. Despite the exclusion of these individuals from analysis, our study had sufficient power to detect the association between the illness and drinking-water (power 98%). High power observed despite the small sample-size was due to the large effect-size and the lower ratio of the non-exposed and exposed individuals. We were not able to isolate *V. cholerae* from the water samples, which was another limitation of our investigation.

As an immediate control measure, the inmates of the shelter-home were supplied with packaged drinking-water, and all the tubewells in the premises were disinfected. For preventing such outbreaks in the future, we recommend (a) relocation of the pond-connected tubewell and colour-coding for the drinking-water tubewells and (b) regular disinfection of the tubewells in the shelter-home.

## ACKNOWLEDGEMENTS

The authors gratefully acknowledge the support provided by the Chief Medical Officer of the Health and District Epidemiological Cell, Howrah district; Director, NICED and Director, STM, Kolkata; Block Medical Officer, Bagnan-I, Howrah; Superintendent of the NGO-run shelter-home and Health Supervisor (male) of Kashmuli Gram Panchayat, Amta-II Block, during the investigation of the outbreak.
